# Unveiling Antimicrobial Properties and Crystallization Induction in PLA Using α‑Ag_2_WO_4_ Nanoparticles

**DOI:** 10.1021/acsapm.3c03012

**Published:** 2024-03-12

**Authors:** Letícia A. Onue, Lara K. Ribeiro, Mariana O. Gonçalves, Elson Longo, Cristina Paiva de Sousa, Marcelo Assis, Sandra A. Cruz

**Affiliations:** † 67828 CDMF, LIEC, Chemistry Department of the Federal University of São Carlos—(UFSCar), São Carlos 13565-905, São Paulo, Brazil; ‡ Morphology and Pathology Department (DMP - UFSCar), Biotechnology Graduation Program (PPGBiotec—UFSCar), São Carlos 13565-905, São Paulo, Brazil; § Department of Analytical and Physical Chemistry, University Jaume I (UJI), Castelló 12071, Spain

**Keywords:** poly(lactic acid), α-Ag_2_WO_4_, composite, crystallization, antimicrobial

## Abstract

In this study, α-Ag_2_WO_4_ nanoparticles were synthesized and then immobilized within the poly­(lactic acid) (PLA) matrix at varying concentrations (0.5, 1.0, and 3.0%). X-ray diffraction revealed the successful incorporation of α-Ag_2_WO_4_ nanoparticles into the PLA matrix without the presence of undesired phases. Furthermore, crystallinity results indicated that the particles did not have the potential to act as nucleating agents under quiescent conditions, but induction in crystallization was observed under nonquiescent conditions. Rheology analysis demonstrated an increase in complex viscosity values for all samples containing α-Ag_2_WO_4_ when compared to those of the pure polymer, indicating good dispersion and distribution. The antimicrobial activity of the composites was particularly effective against and at higher concentrations for and after 16 h of contact. The antimicrobial efficiency was associated with the ability of α-Ag_2_WO_4_ to generate reactive oxygen species (ROS) and the ionic release of Ag^+^, causing irreversible damage to the membranes of these microorganisms. This type of study sheds light on the development of PLA platforms with potential antimicrobial activity and increased crystallization capacity under the employed processing conditions.

## Graphical abstract

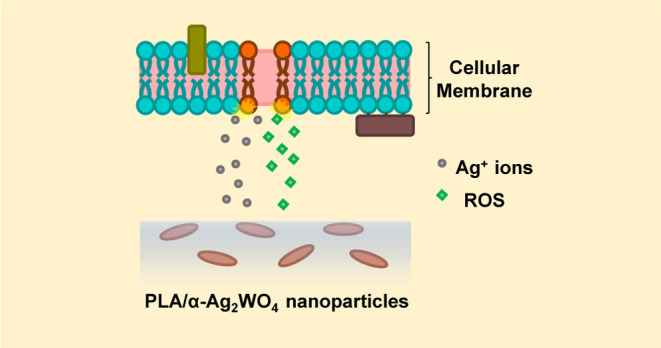

## 1 Introduction

The growing global challenge related to the increase and spread of new diseases highlights the crucial importance of adopting innovative approaches to contain the spread of pathogens.^1^ In this context, the use of antimicrobial plastic materials has emerged as a promising strategy. These plastics, impregnated with antimicrobial agents, demonstrate the ability to inhibit the growth and proliferation of undesirable microorganisms on various surfaces.^2^ This property makes these materials ideal for a variety of applications, from household items and medical equipment to food packaging. The incorporation of antimicrobial plastics not only raises hygiene and safety standards but also plays a fundamental role in reducing the number of contaminations, thus contributing to the preservation of public health.^3^ Investing in technologies that promote antimicrobial resistance in plastics is essential to addressing global health challenges and ensuring safer and more sustainable environments for society.

Polylactic acid (PLA) takes a central position in the current landscape, standing out as a biobased polymer. With its remarkable biodegradability, derived from the monomer obtained from renewable sources such as corn, potatoes, and sugar cane, PLA represents a significant advancement in the pursuit of more sustainable materials.^4,5^ In addition to its direct applications in healthcare, shown to be promising for the development of innovative devices, PLA positively impacts the environment, aligning with a growing global concern about disease spread and the need for antimicrobial materials.^6,7^ Overcoming the peculiarities of PLA, such as its crystallization kinetics and thermal resistance, is crucial to broaden its scope of application.^8^ Studies incorporating inorganic particles, such as talc, clay, and carbon nanotubes, aim not only to overcome these limitations but also to promote the widespread acceptance of this polymer, consolidating it as a fundamental element in the quest for innovative solutions that balance effectiveness, sustainability, and safety across various sectors.^9–11^ Thus, the upward trajectory of PLA not only addresses emerging challenges but also integrates essential contributions to the pursuit of safer and more sustainable environments, directly connecting to the urgent need for innovative approaches in pathogen containment and the preservation of global public health. Some composites utilizing PLA and metal oxides show promise for the development of antimicrobial PLA-based materials. Marra et al. fabricated antimicrobial PLA films with ZnO (1–3% by weight), resulting in significant reductions of () after 48 days of contamination.^12^ In another study, Gonzalez et al. obtained PLA films with TiO_2_ (1–20% by weight) that were effective against biofilms.^13^ In this context, it is crucial to enhance efficiency in terms of antimicrobial action duration and achieve maximum reduction in antimicrobial load. Prolonged contact periods do not offer an efficient solution for antimicrobial proliferation, and high-added loads can profoundly alter the physicochemical properties of the polymer, making its use and processing unfeasible.^14,15^

Among the metal oxides that confer antimicrobial characteristics to various polymers, Ag-based materials have been gaining prominence, including Ag-based semiconductors.^16–18^ Particularly noteworthy in this regard is silver tungstate (α-Ag_2_WO_4_), owing to its versatility, easy fabrication, high stability, and potent antimicrobial activity.^19^ Its robust antimicrobial capacity is attributed to the semiconductor’s ability to generate reactive oxygen species (ROS), even in the dark, and the gradual release of Ag^+^ ions.^20–22^ Pereira et al. successfully produced composite materials comprising α-Ag_2_WO_4_ and chitosan, demonstrating their high antimicrobial efficacy against fungi, bacteria, and viruses.^23^ In another study, Assis et al. developed composites of α-Ag_2_WO_4_ with polypropylene, showcasing the transfer of α-Ag_2_WO_4_ antimicrobial activity to the composite material.^24^ In this context, obtaining composites of α-Ag_2_WO_4_ with a PLA polymeric matrix emerges as an ideal approach for creating new advanced antimicrobial materials. The synergy between α-Ag_2_WO_4_ and PLA holds promising potential for addressing the demand for innovative materials with enhanced antimicrobial properties.

The importance of antimicrobial composite materials, with an emphasis on the prominent role of PLA and Ag-based semiconductors, such as α-Ag_2_WO_4_, is gaining increasing relevance in applications that require antimicrobial efficacy and sustainability. At the core of this approach, this work aims to obtain PLA and α-Ag_2_WO_4_ composites in proportions of 0.5, 1.0, and 3.0 wt %, processed by an internal mixer. The obtained samples were characterized using X-ray diffraction (XRD), differential scanning calorimetry (DSC), thermogravimetry (TGA), and rheometry. Their antimicrobial properties were evaluated through time-kill tests at different intervals, using (), bacteria, and () fungus.

## 2 Experimental Methods

### 2.1 Preparation of α-Ag_2_WO_4_

The synthesis of α-Ag_2_WO_4_ nanoparticles via ultrasound involved the sequential solubilization of key reagents. Sodium tungstate (Na_2_WO_4_·2H_2_O, Strem Chemicals ≥99%) at 1 mmol, silver nitrate (AgNO_3_, Cennabras ≥99.8%) at 2 mmol, and 8.0 mg of citric acid (Sigma-Aldrich ≥99%) were individually dissolved in separate beakers, each containing 100, 70, and 30 mL of distilled water, respectively. Following dissolution, the citric acid solution was introduced into the Ag^+^ ion-containing solution, serving as the stabilizing agent. The resulting solutions of Ag^+^ ions and WO_4_^2–^ were combined to form a 200 mL solution, which underwent ultrasonication using an Eco-sonics washing machine (Q3.0/40A) at a frequency of 40 kHz for 3 h, maintaining a constant temperature of 50 °C. The resulting precipitate was subjected to centrifugation (Centrifuge 5804, Eppendorf) and underwent multiple washes with deionized water to eliminate trace counterions. Finally, the obtained solid was dried in an oven at 60 °C for 10 h. The synthesis scheme is given in Figure S1A.

### 2.2 Preparation of Composites

To minimize the occurrence of degradation by polymer hydrolysis during processing, we previously dried the pristine PLA pellets in a vacuum oven at a constant temperature of 50 °C for 8 h. In Figure S1B, the processing scheme was carried out in an internal mixer (RHEOMIX 600), coupled to a torque rheometer (Thermo Scientific by Haake–Büchler), operating with roller-type rotors, a rotation speed of 50 rpm at a temperature of 210 °C for 4 min. Samples of pristine PLA and PLA containing 0.5, 1, and 3 wt % of α-Ag_2_WO_4_ were prepared. After processing, the material was thermopressed to produce pure PLA and PLA films containing different concentrations of α-Ag_2_WO_4_. The processed samples were previously dried in a vacuum oven at 50 °C for 8 h. The samples were then pressed at a constant temperature of 165 °C in a heat press (TIL Marcon MPH10) for approximately 30 s at 1.0 Torr.

### 2.3 Evaluation of Physicochemical Modification

To analyze the crystal structure of PLA nanocomposite samples, XRD analysis was performed using a Rigaku/MiniFlex 600 (Rigaku, Japan), operating with Cu Kα radiation (1.5406). The diffraction pattern of each sample was obtained between the diffraction angles of 2θ ranging from 5 to 70° at a frequency of 2°/min. The size and shape of the synthesized α-Ag_2_WO_4_ nanoparticles were evaluated using a Supra 35-VP scanning electron microscope (Carl Zeiss, Germany) operating at 5 kV with a secondary electron detector at a distance of 3.5 mm. To determine the effect of the incorporation of α-Ag_2_WO_4_ in the thermal properties of PLA, analyses of DSC were conducted on a DSC NEZSTCH 203 F3-Maia. The thermal properties of the composite samples were obtained by heating from 20 to 200 °C and maintained at that temperature for 3 min, at a scanning rate of 10 °C/min in a flowing nitrogen atmosphere (50 mL/min). Subsequently, they were cooled to 20 °C at 10 °C/min and maintained at that temperature for 1 min. In this step, the influence of α-Ag_2_WO_4_ nanoparticles on the quiescent crystallization of PLA was investigated. Finally, a second heating was performed under the same conditions as those used for the first heating. The composite samples were subjected to TGA analysis to investigate the influence of different concentrations of α-Ag_2_WO_4_ in the degradation process and the thermal stability of the polymer. The analyses were performed on a TGA SDT Q600 (TA Instruments) under the following conditions: heating from 40 to 600 °C at a rate of 20 °C/min in an atmosphere of synthetic air (100 mL/min). Rheological analysis and flow-induced crystallization were performed to verify the influence of α-Ag_2_WO_4_ nanoparticles on the nonquiescent crystallization of PLA. An Anton-Paar (Modular Compact Rheometer, Graz, Austria) MCR 302, equipped with parallel plate geometry with a 25 mm diameter and a 1.0 mm gap, was used. To determine the flow-induced crystallization behavior, the material was melted at 190 °C and the sample was quenched to 115 °C. After the temperature stabilization, a shear rate of 0.1 s^–1^ was imposed on the sample, as the shear tension was simultaneously monitored as a function of time. The time at which the shear tension increases abruptly is defined as the induction time for crystallization.^25^

### 2.4 Antimicrobial Assays

Suspended colonies of ATCC 25922, ATCC 29213, and ATCC 10231, grown for one or two nights on Mueller-Hinton agar plates, were transferred to a test tube containing Mueller-Hinton broth. To standardize the inoculum, colonies were transferred to 0.9% saline until they reached a turbidity of 0.5 on the McFarland scale. Turbidity, expressed as optical density (OD) at λ = 620 nm, corresponded to approximately 1.5 × 10^8^ CFU (colony-forming unit). A 1:10 dilution in 0.9% saline was performed from this solution, establishing an initial test inoculum of 1.0 × 10^7^ CFU/mL. Antimicrobial activity assessment of samples followed the standardized methodology outlined in ISO 21702—Measurement of antibacterial activity on plastics and other nonporous surfaces.^26^ A 100 μL volume of the microbial solution (concentration of 107 CFU/mL) was inoculated in triplicate onto the surface of the samples (2 cm × 2 cm) and covered with a sterile plastic film to ensure even distribution across the tested area. Incubation occurred at 37 °C over four different durations: 2, 4, 8, and 16 h. Following each incubation period, the inoculum was recovered with 10 mL of SCDLP broth, followed by serial dilution in PBS buffer. Each dilution was plated on Mueller-Hinton agar and incubated at 37 °C for 24 h, after which the CFU/cell quantity was determined (see Figure S2).

## 3 Results and Discussion

The inclusion of citric acid in the synthesis of α-Ag_2_WO_4_ intricately influences the kinetics of the nucleation process, modifies the growth rate, and governs the resulting size and morphology of the nanocrystals. The sonochemical synthesis method, involving citric acid, proves to be effective in producing well-defined α-Ag_2_WO_4_ nanocrystals. In the context of forming the polymeric composite, the particle size becomes a critical factor influencing the interaction between the polymer and the particles. The XRD pattern in Figure S3A confirms that the α-Ag_2_WO_4_ nanoparticles possess an orthorhombic structure and an α-phase (ISCD no. 4165), with no discernible presence of secondary phases. Notably, Figure S3B showcases FE-SEM images of α-Ag_2_WO_4_ nanoparticles, exhibiting the rice-like morphology consistent with the findings by Ribeiro et al.

In Figure 1A, XRD was employed to analyze the structural properties of the composites. The PLA employed for α-Ag_2_WO_4_ particle incorporation exhibited polymorphism, capable of crystallizing in seven distinct forms (α, α′, α″, β, γ, ε, and stereocomplex), contingent upon the crystallization conditions.^27^ As observed, all samples exhibited only one peak characteristic of either α′ or α′. The less stable α′ structure, along with other forms (β, γ, and ε), tends to transition to the α phase during heating.^28^ The distinction between the α and α′ phases is evident in the X-ray patterns, with the α′ phase displaying only two characteristic peaks at approximately 16.4 and 18.7°, corresponding to the (200)/(110) and (203) planes, respectively. In contrast, the α phase exhibits these peaks along with others of lesser intensity. Regarding PLA, peaks close to a 2θ angle of 16.2° corresponding to the (200)/(110) planes are also observed in the diffraction. Due to the low intensity of the peak related to the crystal structure plane of the polymer matrix, it was not possible to determine whether this plane corresponds to the α or α′ phase. Considering that the composition of the PLA pellets used in this study (Ingeo 3D450) can incorporate up to 10% by mass of magnesium silicate (talc) according to its safety data sheet, the peaks observed at 8.9, 18.5, and 28.1° are attributed to the (001), (002), and (003) planes, respectively, of the talc crystal structure. In Figure 1B, the magnified XRD pattern provides a clearer visualization of nanocomposite peaks. A small peak near 31.5° is apparent for PLA samples containing α-Ag_2_WO_4_, corresponding to the (231) plane, the most intense peak for the orthorhombic structure of α-Ag_2_WO_4_, as previously depicted in Figure S3A.^29^ No structural alterations were observed in either the PLA matrix or the α-Ag_2_WO_4_ nanoparticles, indicating that composite formation does not alter the intrinsic structure of both materials. It is also possible to observe a change in the color of PLA when adding α-Ag_2_WO_4_ nanoparticles, losing its transparency and resulting in composites with a brown/orange hue (Figure 1C).

**Figure 1:**
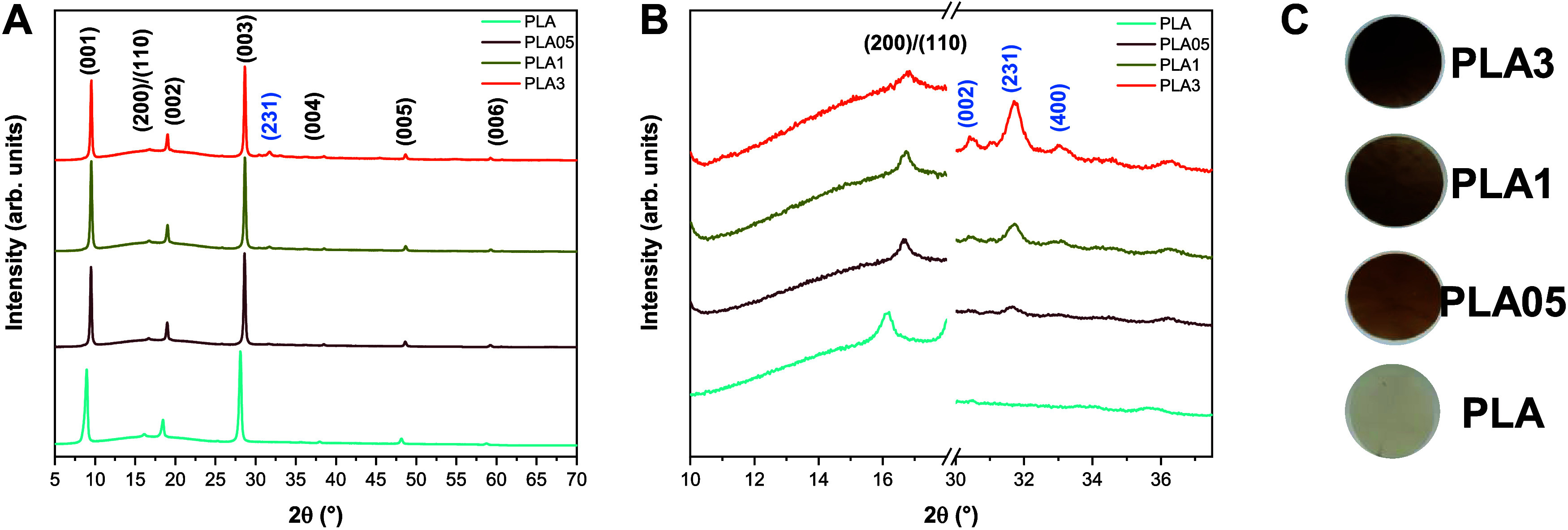
(A) XRD patterns of the PLA/α-Ag_2_WO_4_ composites. (B) The inset of the α-Ag_2_WO_4_ (231) plane. (C) Composites photo after processing.

DSC analysis was used to study the effect of incorporating different concentrations of α-Ag_2_WO_4_ on the thermal properties of PLA. Figure 2A presents the curves referring to the first heating obtained for the samples, and in Table S1, the average and standard deviation of the data extracted in duplicate of the first heating curves are found. It is known that the first heating performed by DSC analysis has an influence on the material processing history in such a way that the conditions used and the process can change the results obtained. However, in general, from the results presented in Table 1, with the incorporation of different concentrations of α-Ag_2_WO_4_ nanoparticles, the glass transition temperature (*T*_g_) and the melting temperature (*T*_m_) remained within the error margin. Between *T*_g_ and *T*_m_, it is possible to observe an exothermic event referring to cold crystallization that occurs during the heating of all samples (see Figure 2A). The cold crystallization temperature (*T*_cc_), which corresponds to the maximum peak of this exothermic event, also remained practically unchanged with the addition of α-Ag_2_WO_4_ nanoparticles regardless of the concentrations used. These results are an indication that the presence of α-Ag_2_WO_4_ nanoparticles does not influence the thermal properties of the polymer under the evaluated conditions.

**Figure 2:**
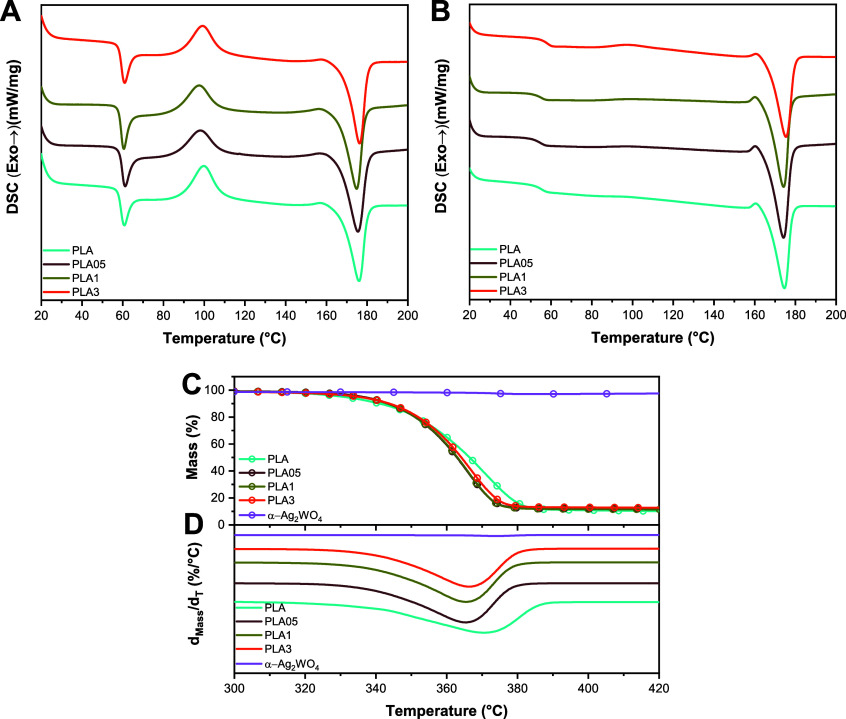
(A) DSC curves for the first and (B) second heating observed for the samples. (C) TG and (D) DTG curves for the samples.

**Table 1:** Thermal Properties of the DSC and TG Curves Observed for the Samples

	PLA	PLA05	PLA1	PLA3
*T*_g_ (°C)	52.40 ± 0.85	52.10 ± 0.57	51.95 ± 0.07	55.05 ± 0.78
*T*_m_ (°C)	174.31 ± 0.41	174.30 ± 0.14	174.25 ± 0.07	175.05 ± 0.49
*T*_cc_ (°C)	160.30 ± 0.14	160.20 ± 0.14	160.15 ± 0.07	160.45 ± 0.07
Δ*H*_m_ (J/g)	24.46 ± 2.50	25.12 ± 0.42	24.02 ± 0.15	22.80 ± 1.11
*T*_i5%_ (°C)	330.8	336.2	335.2	335.1
Δ*T*(°C)	39.9	29.2	30.0	31.1
*T*_max_ (°C)	370.7	365.4	365.2	366.2

In the second heating (Figure 2B), the values obtained for *T*_g_ and *T*_m_, compared to the pristine polymer, did not exhibit changes, except for the sample containing 3% α-Ag_2_WO_4_ nanoparticles, which showed a slight increase due to the restriction of mobility of the amorphous phase containing particles. Furthermore, the change in fusion enthalpy (Δ*H*_m_) from both the first and second heating does not seem to have been altered by the different concentrations of α-Ag_2_WO_4_ nanoparticles. These results support what was deduced during the first heating and demonstrate that the different concentrations of incorporated nanoparticles do not influence the thermal properties of the polymer under the conditions evaluated in this work. It is noteworthy that the results indicate that the presence of particles in the matrix did not cause degradation of the polymer. The presence of the exothermic event related to cold crystallization in the curves of the first heating and its absence in the second heating can be explained by the cooling conditions of the samples. For instance, when a high cooling rate is applied to a molten polymer, there may not be enough time for crystallization to develop. Thus, even if there is the formation of crystalline nuclei, the growth stage will be impaired, especially if the cooling occurs below the *T*_g_, where there is no molecular mobility. When heated after this cooling, the previously formed crystalline nuclei will grow at an accelerated rate (above the *T*_g_), resulting in a rapid recrystallization process known as cold crystallization, as depicted by the curves from the first heating. However, if the cooling conditions of the polymer are controlled to allow for a slower cooling rate, the crystallization process will have time to develop, and upon subsequent heating, the exothermic event related to cold crystallization will not occur.

Still, with regard to the second heating curve, it is possible to observe a small exothermic event (*T*_rc_) before the merger. As expected, the thermogravimetric curves indicate that the obtained α-Ag_2_WO_4_ nanoparticles exhibited high thermal stability and relative purity with no significant solvent contamination, as there was no loss of mass in the temperature range used. TGA was performed to evaluate the thermal stability of PLA/α-Ag_2_WO_4_ nanocomposites by varying the events of mass loss as a function of the temperature. For this work, the initial mass loss temperature (*T*_i_) is considered as the temperature at which the samples lose 5% mass; the maximum mass loss velocity temperature (*T*_max_) is determined by the peak values of the first derivative (DTG) and the difference between *T*_max_ and *T*_i_ (Δ*T*) were obtained through the thermogravimetric curves shown in Figure 2C,D. The TG curves indicate that the α-Ag_2_WO_4_ nanoparticles obtained did not show mass loss in the temperature range used, indicating high thermal stability for the material.

Table 1 presents the parameters described earlier for the composites analyzed under a synthesis air atmosphere. Samples containing α-Ag_2_WO_4_ nanoparticles exhibited higher *T*_i_ values compared to the pristine polymer, indicating that the presence of particles delays the onset of the mass loss process. The strong adhesion between the phases (particle and polymer) reduces the number of defects at the material interface, which, in turn, hinders the release of volatiles.^30^ This behavior is associated with an increase in the free path of volatiles; the higher the nanoparticle content, the slower the volatile diffusion (output of volatiles and input of air atmosphere) through the polymeric mass. Additionally, a higher adhesion between the phases intensifies this effect. This behavior can also be observed by the increase in *T*_g_ (Table 1) of the PLA3 sample. Considering that the peak rate of mass loss occurs near 371 °C for the pristine polymer, the addition of α-Ag_2_WO_4_ nanoparticles to the matrix results in a decrease in *T*_max_, indicating that the presence of nanoparticles accelerates the degradative processes of the matrix. The trends in *T*_i_ and *T*_max_ reflect the kinetics of the mass loss. As observed, the value of Δ*T* (calculated considering 5% initial mass loss) is lower for nanocomposite samples compared to the pure polymer, suggesting that the increase in the rate of mass loss occurs slightly faster due to the presence of the inorganic compound in the matrix. Possible explanations for this effect include (i) the difference in heat capacity between the polymer (organic) and α-Ag_2_WO_4_ nanoparticles (inorganic) and (ii) the distribution of these particles in the matrix. Therefore, as more thermal energy is supplied, these particles heat up faster than the polymer itself, acting as concentration points for this energy and accelerating the degradation processes of the surrounding polymer chains.

As previously described, despite PLA versatility, it is a polymer with low crystallization kinetics. A change in this characteristic is desirable using a second phase, as is the case with α-Ag_2_WO_4_ nanoparticles. Crystallization conditions (quiescent and nonquiescent) can significantly influence the process. One way to investigate the nucleating effect of a particle in a composite is by calculating the degree of crystallinity (Table 1) and the crystallization temperature (*T*_c_) under quiescent conditions. The degree of crystallinity is the percentage of the crystalline portion of a semicrystalline substance, which can be determined by eq 1

1 $$X_{c}=\frac{\left(\Delta H_{m}-\Delta H_{\mathrm{cc}}\right)}{\left(1-\varphi \right)\Delta H_{0}}100\%$$

where φ is the mass percentage of the nucleating agent and Δ*H*_0_ is the melting enthalpy of perfectly crystallized PLA, which is 93.6 J/g. The subtraction of the enthalpy of cold crystallization (Δ*H*_cc_) is applied when the degree of crystallinity of the material is evaluated resulting from its processing history. This subtraction was applied in the crystallinity calculation for the results obtained in the first heating and disregarded in the second heating. Both the degree of crystallinity (*X*_c_) in the first heating and the second heating, calculated from the Δ*H*_m_ values of their respective curves, did not show a significant variation with an increase in the concentration of the α-Ag_2_WO_4_ nanoparticles. The *X*_c_ values obtained in the first heating are smaller in magnitude than those presented in the second heating, implying that processing affects the crystallization of the samples, resulting in a less crystalline material. Furthermore, considering the *X*_c_ results of the second heating, it can be inferred that the presence of the particle does not influence the dynamic and quiescent crystallization of the polymer.

Figure S4 refers to the cooling step performed between the first and second heatings of the pure PLA and film samples. Despite the low crystallization kinetics inherent in PLA, the cooling rate used and the presence of talc in the polymer composition allowed the quiescent crystallization process to develop during the cooling step in all samples. Furthermore, it is observed from the results in Figure 3A that there is no change in the crystallization temperature (*T*_c_) values, indicating that the particle under quiescent conditions does not alter the crystallization kinetics of this polymer. Figure 3B shows the stress (τ) as a function of time under quiescent conditions at a shear rate of 0.1 s^–1^.

**Figure 3:**
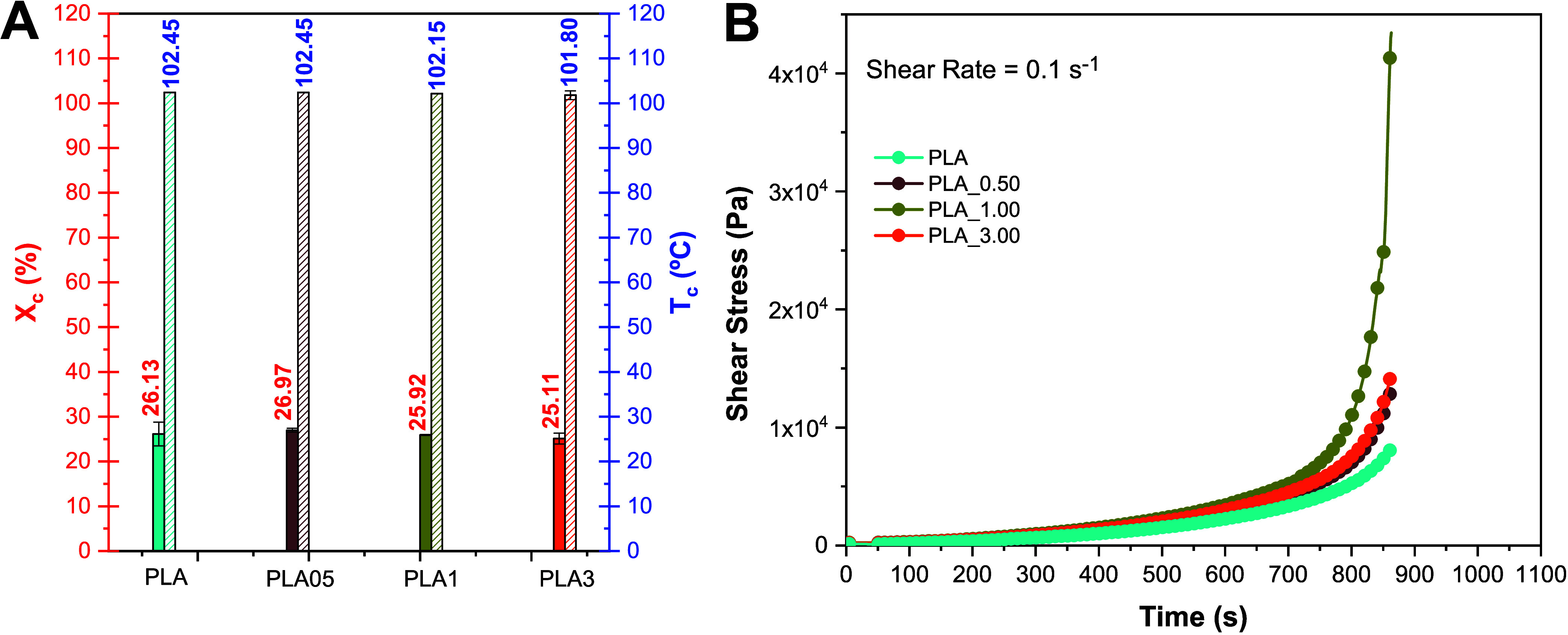
(A) Nonquiescent conditions: crystallinity rate and crystallization temperature for the samples. (B) stress (τ) under quiescent (shear rate of 0.1 s^–1^).

During the initial approximately 300 s of analysis, the shear stress remains constant, indicating constant viscosity. However, after a certain period, the stress sharply increases (tending toward infinite viscosity). In other words, initially, the samples exhibit viscous behavior in the molten state (Newtonian fluid), and after a certain duration, this behavior transitions to a solid state.^31^ The presence of α-Ag_2_WO_4_ nanoparticles influences the induction time for the onset of flow-induced crystallization in all samples at a shear rate of 0.1 s^–1^. Nanoparticles are known to impact the crystallization process of a semicrystalline polymer.^32^ During the nucleation step, nanoparticles can serve as nucleating agents by reducing the surface energy to form critical radius nuclei. Although the α-Ag_2_WO_4_ nanoparticles may not have demonstrated the potential to act as a nucleating agent under quiescent and dynamic cooling conditions, as indicated by the DSC data, when a shear rate (0.1 s^–1^) is applied, the various nanoparticle concentrations come into play during the isothermal and nonquiescent crystallization process, accelerating it by reducing the induction time. Furthermore, there appears to be an optimum concentration (in this case, 1%). Lower concentrations do not induce crystallization, and higher concentrations can hinder the process. Additionally, the induction time increases when 3% of α-Ag_2_WO_4_ nanoparticles is incorporated into the matrix at a rate of 0.1 s^–1^. With a larger quantity of nanoparticles added, the diffusion of the chains in the growth step is significantly affected and the nanoparticles start to act as a physical barrier, reducing the mobility of the chains. Consequently, the crystallization kinetics is affected, leading to an increase in the induction time.

Initially, a linear viscoelasticity analysis was performed to determine the level of deformation to be used in subsequent rheometric analyses so that the molecular structure of the samples was not compromised. Under an oscillatory shear regime, the stress (τ) or strain (γ) applied to the polymer varies with the imposed frequency (ω). Therefore, the stress or strain amplitude must be small enough to ensure that measurements remain within the linear viscoelasticity regime. As polymers are viscoelastic, subjected to a small-amplitude shear strain (or stress) level, stresses (or strains) will oscillate with the same frequency but will not be in phase with the stress (or strain), which presents linear viscoelastic behavior. For this work, a deformation level corresponding to 1% was selected for the rheological tests. Figure 4A presents the complex viscosity curves (η*) as a function of the angular frequency (ω) for the samples. From the angular frequency scan analysis, it is possible to obtain information about the complex viscosity of a material. Complex viscosity values as a function of angular frequency for nanocomposite materials can provide information on the interaction between its components (polymer and inorganic particle). By this technique, the polymeric chains are subjected to a stretching imposed by the shear rate under increasing angular frequency. Macromolecular chains, when stretched by the imposed conditions, tend to return to their thermodynamically more stable state, the entangled state. When the imposed angular frequency is low, the stretching of these chains is minimal, and what is observed under these conditions is the interaction between the components of the sample (such as the nanoparticle and the polymer in the case of nanocomposite material). This rheological response is visualized by changes in complex viscosity values when lower angular frequencies are applied.

**Figure 4:**
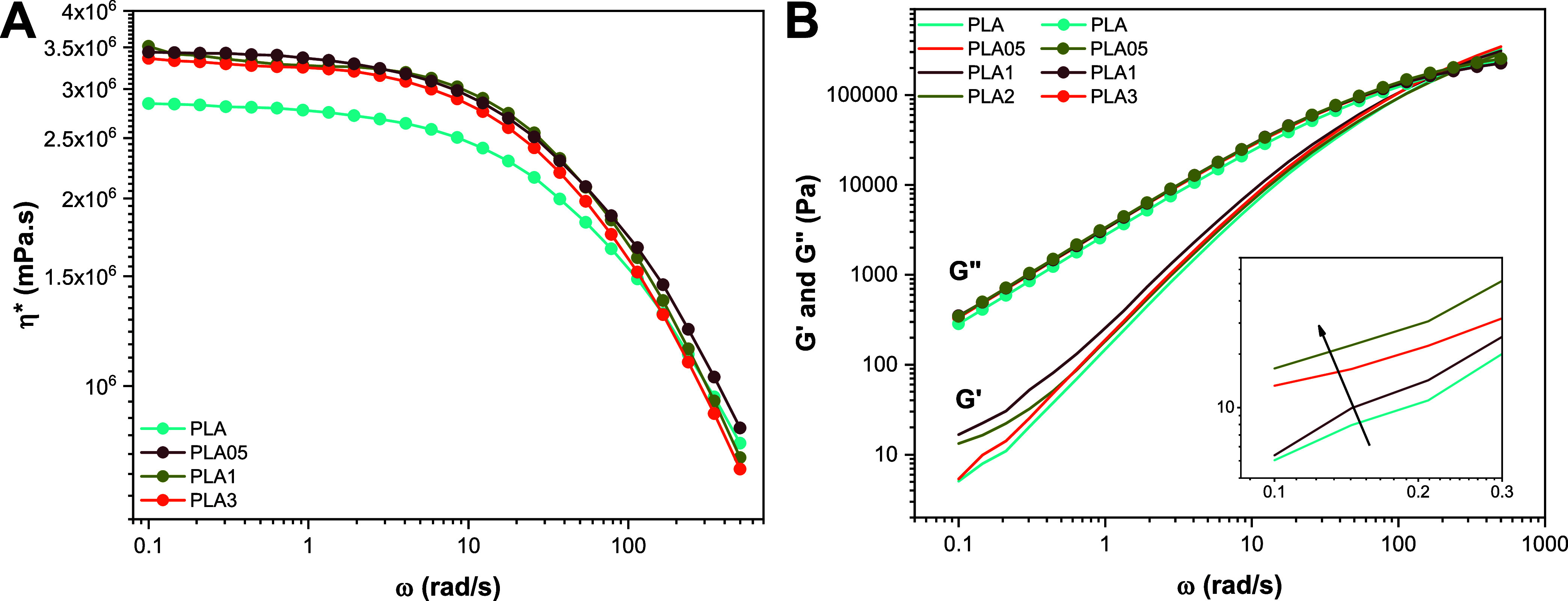
(A) Complex viscosity as a function of frequency for the samples. (B) Storage (*G*′) and loss modulus (*G*″) as functions of the sample frequencies.

In the region of lower frequencies, there was an increase in complex viscosity values for all samples containing α-Ag_2_WO_4_ nanoparticles compared with the pure polymer. As indicated by Sadeghipour et al.^33^ in binary systems (polymer-nanofiller), the increase in viscosity is related to good dispersion and charge distribution, and higher initial values of η* may indicate greater interaction between nanoparticles and the polymer matrix. In the high-frequency region, the complex viscosity curves for all samples tend to converge. This occurs because the interaction between the particle and the polymer is very small in relation to the influence of the high angular frequency on the behavior of the material under these conditions. Therefore, the rheological response observed in this region is predominantly given by the molecular structure of the matrix, i.e., the polymer. Then, there is a separation between the curves in this region of higher frequencies; this is an indication that there has been a change in the macromolecular structure as a result of the degradation of the material. However, in Figure 4A, the convergence between the curves indicates that the polymer was not degraded with the incorporation of different concentrations of α-Ag_2_WO_4_ nanoparticles, which implies that the presence of particles did not induce PLA degradation, corroborating the results of DSC and TGA previously presented in such a way that the lower value of Δ*T* observed in the thermogravimetry analysis is not related to the low adhesion between the polymer and the load or the direct effect of the particle on the degradation process but rather with the difference in the heat capacity of each material.

Figure 4B presents the storage (*G*′) and loss modulus (*G*″) curves as a function of angular frequency (ω). The term *G*′ is associated with the elastic component or with the storage of energy in each cycle, while the term *G*″ is associated with the component out of phase with the deformation; that is, it is related to the viscous contribution or energy dissipation. Table 2 presents the values of the angular coefficient (α) of the curves of *G*′(ω) and *G*″(ω) in the region corresponding to the interval between 0.1 and 0.44 rad/s of the angular frequency, that is, in the terminal zone. The *G*′ becomes greater than *G*″ in the region of higher frequencies, indicating the typical behavior of a viscoelastic solid. On the other hand, in the region of lower frequencies, where *G*″ > *G*′, there is the behavior of a viscoelastic fluid. Furthermore, as the number of nanoparticles is added, an increase of *G*′ is observed in the low-frequency regions, which may indicate a strong interaction between the nanoparticles and PLA.^34^ As observed by Liu et al.,^35^ an increase in the concentration of embedded nanoparticles can affect the dynamics of the relaxation of polymeric chains, slowing this process and increasing the elasticity of the material (observed by the substantial increase in *G*′(ω)).

**Table 2:** Slope (α) of the *G*′ and *G*″ Curves for Frequency (ω) for the PLA and PLA/α-Ag_2_WO_4_ Film Samples

	curve *G*′(ω^α^)		curve *G*″ (ω^α^)	
samples	*R* ^2^	α	*R* ^2^	α
PLA	0.980	1.342 ± 0.0946	1	0.989 ± 0.000652
PLA05	0.991	1.441 ± 0.0703	0.999	0.993 ± 0.00129
PLA1	0.980	1.089 ± 0.0775	0.999	0.964 ± 0.00595
PLA3	0.974	0.912 ± 0.0737	1	0.982 ± 0.000989

It is also possible to investigate whether there was the formation of a percolated network through the rheometric data in an oscillatory regime.^36^ Monitoring the variation of slopes in the terminal zone (when ω → 0) of the functions *G*′(ω) and *G*″(ω) allows evaluation of the viscoelastic behavior of the nanocomposite. For example, in pure melt polymers, the slope of the curve *G*′(ω) is equal to 2 (which means that *G*′ is proportional to ω2), and the slope of the curve *G*″(ω) is equal to 1 (where *G*′ is proportional to ω) in the terminal zone. In polymers that exhibit solid behavior, the slope of the curve *G*′(ω) is equal to zero. Thus, when there is a decrease in the slope of these curves, a nanocomposite material (containing nanoparticles) starts to behave similarly to a solid material, meaning that the addition of small proportions of nanoparticles causes the pure molten polymer to pass from a liquid to a solid state.

After conducting an analysis of the physicochemical properties of composite materials, their potential applications as antimicrobial substances were assessed. The objective was to confirm that the antimicrobial activity of α-Ag_2_WO_4_ nanoparticles would be retained in the composites with PLA. To evaluate the antimicrobial potential of PLA/α-Ag_2_WO_4_ developed in this study on film surfaces, antimicrobial assays were performed against various microorganisms (, , and ) at different contact intervals with the nanocomposite surface (2, 4, 8, and 16 h). During the 16 h incubation and growth period, in the tests for (Figure 5A), the pure PLA sample exhibited a 27% inhibition variation compared to the initial hours of contact with the bacteria. Nanocomposite samples demonstrated a remarkable 99% reduction in levels compared to PLA after incubation, highlighting its efficacy against this bacterium. For (Figure 5B), a similar behavior was observed between the PLA sample and the nanocomposites at all times. However, after 16 h, a roughly 3 times greater elimination of composites was observed compared to pure PLA. This distinctive behavior toward both bacteria is attributed to Gram-positive bacteria () differing from Gram-negative bacteria () in their elimination capacities upon contact with various materials.^37^ The Gram-positive bacteria possess a cell wall characterized by numerous interconnected layers of peptidoglycan, creating a substantial and sturdy structure. This is in sharp contrast to Gram-negative bacteria, which feature only a thin layer of peptidoglycan. Regarding , a significantly greater reduction was observed after 16 h of contact for the sample with 3% α-Ag_2_WO_4_ nanoparticles (Figure 5C). The cell wall of fungi is more complex than that of bacterial membranes, and this complexity can lead to greater resistance to certain antimicrobial treatments. In this sense, it is observed that the antimicrobial activity of PLA is dose-dependent on the concentration of α-Ag_2_WO_4_, a result already evidenced in previous studies where the minimum inhibitory concentration of these nanoparticles in their powder form was evaluated for , , , and even the SARS-CoV-2 virus.

**Figure 5:**
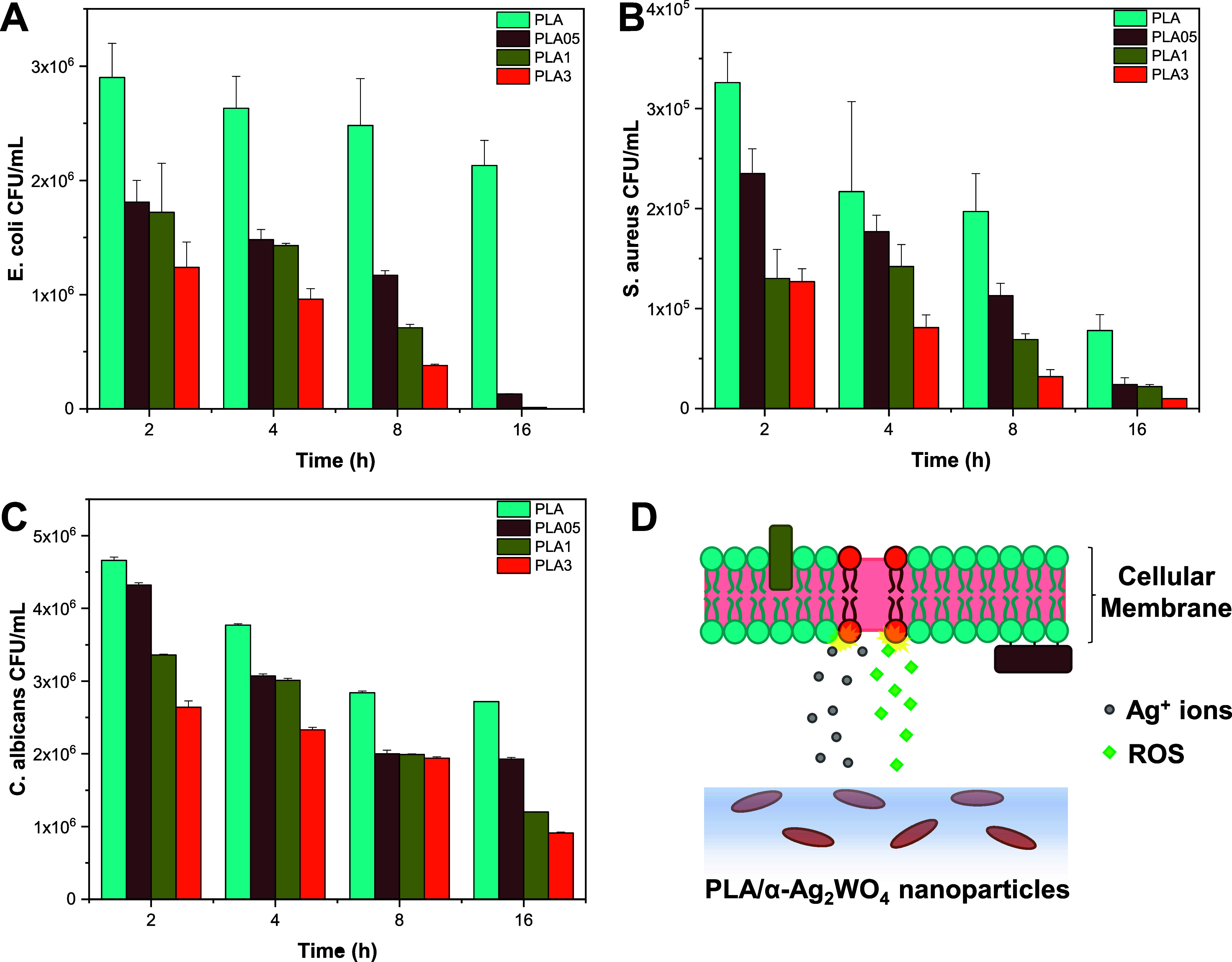
Time-kill results for (A) , (B) , and (C) . (D) Proposed mechanism of the PLA/ α-Ag_2_WO_4_ composites.

The results indicate that the composites synthesized here possess the ability to inactivate and/or significantly reduce the growth of pathogens within a short time interval, depending on the nature of the microorganism, the exposure time, and the concentration of α-Ag_2_WO_4_ nanoparticles. Within the band gap of α-Ag_2_WO_4_, it is feasible to convert oxygen molecules (O_2_) into superoxide radicals (^•^O_2_^–^) via electrons (e^–^) and oxidize water molecules (H_2_O) to hydroxyl radicals (^•^OH) and a proton (H^+^) via holes (h^+^). This H^+^ can further react with ^•^O_2_^–^ to form the hydroperoxyl radical (^•^OOH). These radicals collectively form ROS that can swiftly interact with the membrane components of pathogenic microorganisms.^38^ This interaction induces severe enzymatic and structural alterations, ultimately leading to the demise of the microorganisms. Additionally, this material can lead to the release of Ag^+^ ions, which can interact irreversibly with intracellular components, causing a loss of function and, occasionally, cell death.^39^ These results are in accordance with previous studies where the antimicrobial activity of α-Ag_2_WO_4_ particles was evaluated.^21,22,39–41^ The schematic representation of the action mechanism of nanocomposites is depicted in Figure 5D.

## 4 Conclusions

PLA is renowned for its favorable attributes, including biodegradability and biocompatibility. Nonetheless, certain limitations, such as sluggish crystallization kinetics and a lack of inherent antimicrobial activity, hinder its broader applicability. The crystallinity results suggested that the α-Ag_2_WO_4_ lacked the potential to function as nucleating agents under quiescent conditions on PLA. However, induction of crystallization was observed when nonquiescent conditions were applied. This property can open possibilities for various applications, especially those related to injection molding and additive manufacturing, once these processes occur under a shear flow. These alterations give rise to enhanced characteristics within the matrix, previously undocumented in the literature. An additional favorable aspect is evident through rheological analysis, which demonstrated an increase in complex viscosity values for all samples containing α-Ag_2_WO_4_ compared to the pure polymer. This indicates an effective dispersion and distribution of the particles in the polymer matrix. The composites exhibited notable antimicrobial effectiveness, especially against , with increased efficacy observed at higher concentrations for and after 16 h of contact. The antimicrobial efficiency was attributed to the α-Ag_2_WO_4_ capability to generate ROS and the release of Ag^+^ ions, leading to irreversible damage to the membranes of these microorganisms. In conclusion, the composite multifunctional nature positions it as a material capable of meeting the diverse needs of industries, such as food packaging and medical devices. Its unique combination of properties makes it not only versatile but also an innovative solution for addressing the evolving challenges in these sectors.

## Supplementary Material



## Notes

Thermal properties from the DSC curves, DSC crystallization curves, and XRD and SEM images of the α-Ag_2_WO_4_ nanoparticles (PDF)

## Abbreviations

(PLA): poly­(lactic acid)
(α-Ag_2_WO_4_): silver tungstate
(XRD): X-ray diffraction
(TGA): thermogravimetric analysis
(PBS): phosphate buffer saline
(DSC): differential scanning calorimetry
(*T*_g_): glass transition temperature
(*T*_m_): melting temperature
(*T*_cc_): cold crystallization temperature
(Δ*H*_m_): fusion enthalpy
(T_i_): initial mass loss temperature
(*T*_max_): maximum mass loss velocity temperature
(Δ*H*_cc_): cold crystallization
(*X*_c_): degree of crystallinity
(Tc): crystallization temperature
(*G*′): storage modulus
(*G*″): loss modulus
(η*): complex viscosity
(τ): stress
(γ): strain
CFU: (colony-forming unit)
(ROS): reactive oxygen species
(e^–^): electrons
(^•^O_2_^–^): superoxide radical
(^•^OH): hydroxyl radical
(H^+^): proton
(^•^OOH): hydroperoxyl radical
(h^+^): holes
