# Supplementary material for: Unveiling Antimicrobial Properties and Crystallization Induction in PLA Using α‑Ag2WO4 Nanoparticles
Source: ACS Appl Polym Mater. 2024 Mar 12;6(6):3233–42. doi: 10.1021/acsapm.3c03012 (PMC12320826; doi:10.1021/acsapm.3c03012)
Supplement: Supplementary file 1 [file ap3c03012_si_001.pdf]

SUPPORTING INFORMATION

Unveiling Antimicrobial Properties and Crystallization Induction in PLA Using α-Ag_2WO_4 Nanoparticles

Letícia A. Onue[1, †], Lara K. Ribeiro[1, †], Mariana O. Gonçalves[2], Elson Longo[1], Cristina Paiva de Sousa[2], Marcelo Assis[3], Sandra A. Cruz[1*]*

[1]CDMF, LIEC, Chemistry Department of the Federal University of São Carlos – (UFSCar) São Carlos 13565-905, SP, Brazil

[2]Morphology and Pathology Department (DMP - UFSCar), Biotechnology Graduation Program (PPGBiotec – UFSCar) São Carlos 13565-905, SP, Brazil

[3]Department of Analytical and Physical Chemistry, University Jaume I (UJI), Castelló 12071, Spain

[†] The authors played equally significant roles in the advancement of this work.

* Corresponding author: marcelostassis@gmail.com; sandra.cruz@ufscar.br

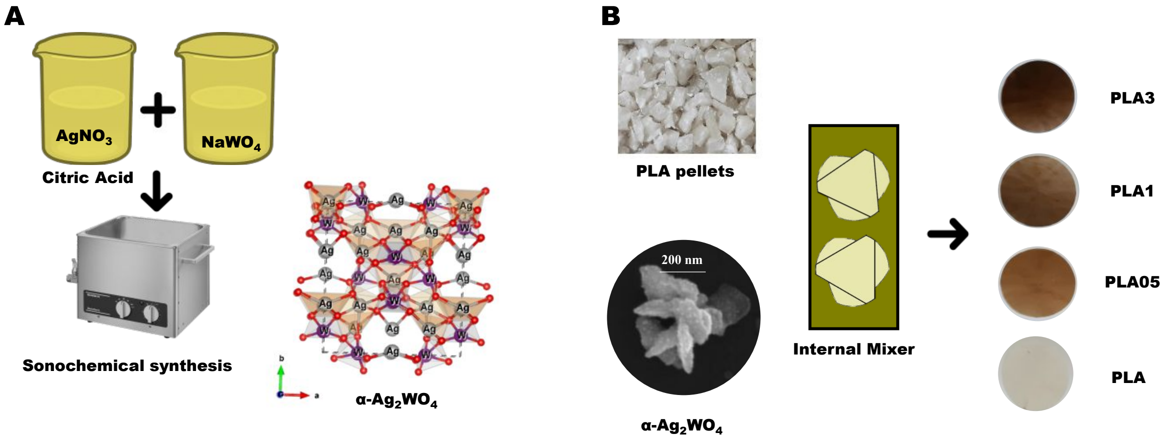

Figure S1. **A)** Synthesis scheme of α-Ag_2WO_4 by sonochemical synthesis. **B)** Processing the mixture of PLA pellets and α-Ag_2WO_4 to form PLA/α-Ag_2WO_4 composites.

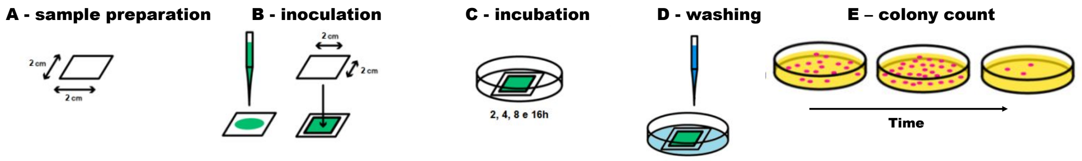

Figure S2. (A-E) Schematic representation of the antimicrobial test according to the ISO 22196 standard.

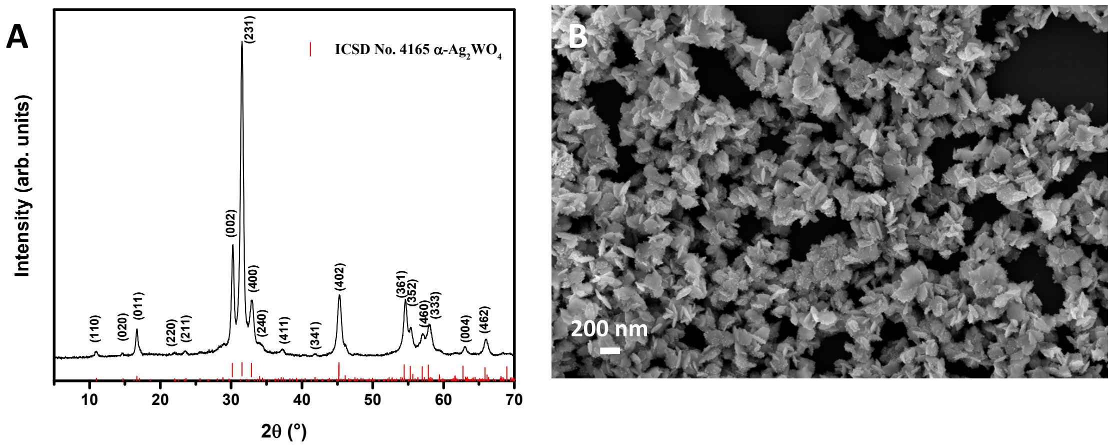

Figure S3. A) XRD and **B)** SEM images of the α-Ag_2WO_4 nanoparticles.

Table S1. Thermal properties from the DSC curves for the first heating observed for the PLA and PLA/α-Ag_2WO_4 nanocomposite samples.

	**PLA**	**PLA_0.50**	**PLA_1.00**	**PLA_3.00**
**Tg (°C)**	57.95 ± 0.21	58.55 ± 0.07	57.80 ± 0.28	57.55 ± 0.78
**Tcc (°C)**	98.80 ± 1.41	98.70 ± 0.71	97.65 ± 0.07	99.35 ± 0.07
**Tm (°C)**	175.75 ± 0.49	175.45 ± 0.07	175.00 ± 0.28	175.80 ± 0.71
**ΔHcc (J/g)**	15.20 ± 1.42	13.70 ± 1.36	10.18 ± 4.01	10.30 ± 5.01
**ΔHm (J/g)**	26.85 ± 1.69	27.89 ± 0.95	25.32 ± 0.71	24.18 ± 2.19
**X_c (%)**	12.45 ± 3.32	15.24 ± 0.44	16.34 ± 5.09	15.27 ± 3.91

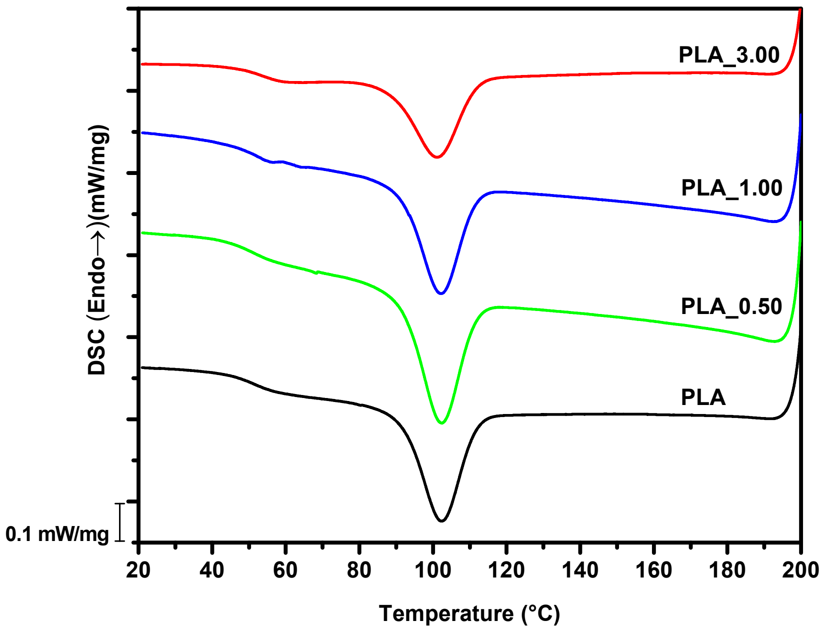

Figure S4. DSC crystallization curves of samples.
